# Cerebral Intraparenchymal Hemorrhage Changes Patients’ Gut Bacteria Composition and Function

**DOI:** 10.3389/fcimb.2022.829491

**Published:** 2022-03-16

**Authors:** Zujian Xiong, Kang Peng, Shaoyu Song, Yongwei Zhu, Jia Gu, Chunhai Huang, Xuejun Li

**Affiliations:** ^1^ Department of Neurosurgery, Xiangya Hospital, Central South University, Changsha, China; ^2^ Hunan International Scientific and Technological Cooperation Base of Brain Tumor Research, Xiangya Hospital, Central South University, Changsha, China; ^3^ Xiangya School of Medicine, Central South University, Changsha, China; ^4^ Department of Neurosurgery, First Affiliated Hospital of Jishou University, Jishou, China; ^5^ Centre for Clinical and Translational Medicine Research, Jishou University, Jishou, China

**Keywords:** gut bacteria, cerebral intraparenchymal hemorrhage, metagenomic shot sequencing, function annotation, single-species analysis

## Abstract

Gut bacteria consists of 150 times more genes than humans that are vital for health. Several studies revealed that gut bacteria are associated with disease status and influence human behavior and mentality. Whether human brain injury alters the gut bacteria is yet unclear, we tested 20 fecal samples from patients with cerebral intraparenchymal hemorrhage and corresponding healthy controls through metagenomic shotgun sequencing. The composition of patients’ gut bacteria changed significantly at the phylum level; Verrucomicrobiota was the specific phylum colonized in the patients’ gut. The functional alteration was observed in the patients’ gut bacteria, including high metabolic activity for nutrients or neuroactive compounds, strong antibiotic resistance, and less virulence factor diversity. The changes in the transcription and metabolism of differential species were more evident than those of the non-differential species between groups, which is the primary factor contributing to the functional alteration of patients with cerebral intraparenchymal hemorrhage.

## Introduction

The gut microbiota stability plays a pivotal role in maintaining the host’s homeostasis and brain development (Carlson et al., 2018; Bolte et al., 2022). Through Influencing the balance between bacterial anti-inflammatory and pro-inflammatory properties, dysbiosis contributes to inflammation and various disease severity, leading to a worse clinical outcome. (Tilg et al., 2020; Zuo et al., 2020; Gou et al., 2021; Szychowiak et al., 2022). The composition of the gut microbiota is influenced by various factors, including environment, host disease state, host immune response and genetic background (Wu et al., 2020; Gou et al., 2021), among which the environment is a critical factor in gut bacterial property transformation. Since the communication between the gut and the brain is bi-directional, the fecal microbiome from patients with chronic traumatic brain injury changed differently (Urban et al., 2020) and the changed gut microbiota will subsequently have profound impacts in influencing the host’s neurological function and behaviors, simultaneously affecting neurodegeneration and the repair process post-neurological trauma (Cryan and Dinan, 2012; Sampson and Mazmanian, 2015). For example, several studies reported that the gut microbiota affects the outcome of acute brain injury in mice by regulating the immune system (Denes et al., 2015; Benakis et al., 2016). In addition to the immune system that connects the gut and the brain, the vagus nerve provides a direct connection between the central nervous system and the enteric nervous system during gut bacteria-brain interaction, allowing the gut microbiota to send microbial signals from the gastrointestinal tract directly to the brain (Forsythe et al., 2014). Metabolism is a leading mechanism for the gut microbiota to influence brain function as it is involved in numerous aspects of the metabolism process: from producing metabolic precursors for the hormones and neurotransmitter metabolism to directly producing the active metabolites, such as acetate, a short-chain fatty acid that can cross the blood-brain barrier and reduce appetite (Frost et al., 2014; Lyte, 2014; Sharon et al., 2014; Jameson et al., 2020; Chen et al., 2021).

Cerebral intraparenchymal hemorrhage (IPH) has a higher incidence in the Asian and older populations than the others. It also exerts the highest mortality and substantial morbidity among all forms of stroke (Gross et al., 2019). Houlden et al. revealed that acute brain injury induces gut microbiota dysbiosis in mice due to increased noradrenaline release from the autonomic nervous system into the gut (Houlden et al., 2016). However, whether brain injury, especially the IPH, will change the human gut microbiota composition or function is yet unknown, and taxonomic and functional profiling is required to elucidate the gut microbiota (Schmidt et al., 2018). Nowadays, metagenomic shotgun sequencing provides a powerful tool to accurately detect microbiota and predict microbial biological features compared to 16S amplicon sequencing (Weinstock, 2012). Herein, we performed metagenomic shotgun sequencing on 20 human fecal samples (10 cases and 10 controls) at the same region and the same period to explore the gut microbiota composition changes associated with IPH. The functional changes characterizing the IPH patients’ gut microbiota were determined by various databases. We also identified the group-specific species that altered after IPH occurred and explored the functional differences among these species.

## Materials and Methods

### Study Cohort and Patient Characteristics

The 20 fecal samples, including stools from 10 operation-free patients who suffer from cerebral intraparenchymal hemorrhage within 7 days and 10 healthy individuals as the control group, were collected from the First Affiliated Hospital of Jishou University, Hunan, China (**Table S1**). Written informed consent was obtained from all participants. Diagnosis was established on the Guidelines for Multidisciplinary Diagnosis and Treatment of Hypertensive Cerebral Hemorrhage in China (2020) (Chinese Medical Association Neurosurgery Branch et al., 2020). The patients with cerebral IPH caused by blood disease, aneurysm, vascular malformation, and liver disease were excluded from the study. The healthy controls had no history of hypertension or diabetes. Moreover, none of the participants had any history of bowel disease, antibiotic use, or usage of drugs affecting bowel function in the past 3 months. The study conformed to the ethical guidelines of the 1975 Declaration of Helsinki and was approved by the Institutional Review Board of Jishou University.

### Fecal Sample Collection and DNA Extraction

Fecal samples were freshly collected from each participant and frozen at −80°C. The DNA was extracted using Longseegen Mini Stool DNA Isolation kit, according to the manufacturer’s recommendation and quantified by agarose gel electrophoresis and Qubit Fluorometer.

### Metagenomic Sequencing and Data Processing

The paired-end sequencing was performed on the Illumina HiSeq platform (paired-end library 400 bp and read length 150 bp). After quality control, including removal of adaptors and low-quality reads by Fastp (version 0.20.1, parameter: -n 3 -q 20 -u 50 -l 30 -c) (Chen et al., 2018), the host DNA reads were removed by Bowtie2 (version 2.4.2) (Langmead and Salzberg, 2012) using GRCh38 genome assembly as reference. On average, 14.9 (11–20) Gbp of high-quality non-host sequences were obtained for each sample, and then the remaining microbial reads were filtered by Khmer (Crusoe et al., 2015) and aligned to the Unified Human Gastrointestinal Genome (UHGG) database by Kraken2 (version 2.0.8) and Bracken for taxonomic annotation with default parameters (Lu et al., 2017; Wood et al., 2019; Almeida et al., 2021).

For each sample, we used megahit (version 1.2.9) with a series of k-mer values (21–61, step=4) to assemble the reads into contigs (Li et al., 2015) and choose the optimal k-mer (k=33) with the most reads >1000 bp by quast evaluation (Gurevich et al., 2013). Bacterial genes were predicted on contigs longer than 500 bp using Prokka (version 1.14.6) (Seemann, 2014).

### Diversity and Rarefaction Curve

To evaluate the richness and diversity of bacteria in each sample, we calculated the within-sample α-diversity using Chao 1 and Shannon indexes, respectively. The inter-sample β-diversity was evaluated by the weighted unifrac distance and further processed by PCoA in the ape R package (Lozupone and Knight, 2005; Paradis and Schliep, 2019).

Rarefaction analysis was conducted to evaluate the species richness. We performed random sampling 20 times with step=20000 to estimate the total number of species from these samples by the vegan R package.

### Group-Specific Species Identification

Next, we performed linear discriminant analysis (LDA) effect size (LEfSe) analysis to identify specific species between the patient and healthy control groups based on Kruskal–Wallis rank-sum test, Wilcoxon rank-sum test, and linear discriminant analysis (LDA) score (Segata et al., 2011). lgLDA >2 indicates the specificity of the species.

On the other hand, MaAslin analysis was conducted to calculate the correlation strength of each species with groups (Morgan et al., 2012). The species with co-efficient >0 and false discovery rate (FDR) <0.05 were selected as the group-related species.

### Functional Annotation

All genes were aligned to the Evolutionary Genealogy of Genes: Non-supervised Orthologous Groups (eggNOG) 5.0 database using eggnog-mapper v2 with default parameters (Huerta-Cepas et al., 2017; Huerta-Cepas et al., 2019). The results of eggnog-mapper also consisted of the Kyoto Encyclopedia of Genes and Genomes (KEGG) orthologs, pathways, modules, and Clusters of Orthologous Groups (COG) functional catalogs. The annotation and hierarchical correlation within the KEGG pathways were downloaded from the KEGG database (https://www.genome.jp/kegg/). The statistics of enrichment catalogs and pathways were calculated through relative abundance (the catalog frequency in each sample/total catalog frequency of each sample). The reference for Carbohydrate-Active Enzymes (CAZy) was downloaded from http://www.cazy.org/ (Lombard et al., 2014), and the CAZy reads of each sample were identified by hmmer (version 3.1, e-value cutoff=1e-05) (Mistry et al., 2013). The relative abundance of the total CAZy reads was calculated in count per million (CPM, (CAZy reads per sample×1e06)/total non-host reads per sample) and compared using the Wilcoxon rank-sum test. Subsequently, the enzymes with FDR <0.05 were selected and annotated into specific KEGG pathways as shown in **Figure S2**. The virulence factors secreted by bacteria were identified using Basic Local Alignment Search Tool (BLAST) to align non-host reads to the virulence factor sequence reference downloaded from the Virulence Factor Database (VFDB) with e-value cutoff at 1e-05 (Liu et al., 2019). The identified virulence factor reads were also used to analyze the relative abundance (CPM) further. The different virulence factors between groups were selected by the Wilcoxon rank-sum test (FDR <0.05), following which the selected virulence factors in specific structures or species were counted (**Figure S2**). The antibiotic resistance genes were identified by ariba against the reference sequence downloaded from the Comprehensive Antibiotic Resistance Database (CARD) (assemble threshold =0.97) (McArthur et al., 2013; Hunt et al., 2017). The antibiotic-resistant genes were normalized in CPM and selected based on the Wilcoxon rank-sum test with FDR <0.05. To explore the difference in bacterial secondary metabolites between groups, we predicted the secretion of such metabolites by Antismash (version 5.1.2) based on non-host reads of each sample (Blin et al., 2019).

### Functional Modules Predicted From Metagenomics

All KEGG orthologs identified in metagenomic functional annotation were enriched in the modules or pathways by Omixer-RPM (version 1.1, coverage=1) were based on the previously published KEGG metabolic module and gut-brain module (GBMs) sets (Darzi et al., 2016; Valles-Colomer et al., 2019). The different modules between groups were compared using the limma R package; those with logFC >2 and FDR <0.05 were considered as group-specific modules.

### Hub Bacteria Identification by the Weighted Network Analysis

A bacterial weighted correlation network was constructed using WGCNA R package (Langfelder and Horvath, 2008). The signed correlation network was constructed based on the relative taxonomic abundance of the species obtained by Kraken2 analysis. The adjacency matrix was created by Pearson’s correlation analysis on the species taxonomic table with 14 as the soft threshold (**Figure S5B**). Then, a topological matrix was built using the topological overlap measure (TOM), an advanced co-expression measure that considered the correlation between two species and the extent of their shared correlations across the weighted network (Zhang and Horvath, 2005; Li and Horvath, 2007; Yip and Horvath, 2007). Finally, we chose the dynamic hybrid cut method, a bottom-up algorithm, to identify the correlation bacteria modules based on their topological overlap matrix (Langfelder et al., 2008). The modules with <50 species were filtered out. To identify the significance of each module, species significance was calculated and the correlation between the species and groups assessed. Module significance (MS) was defined as the average species significance within modules and calculated to measure the correlation between the modules and groups (Ghazalpour et al., 2006). Statistical significance was determined using the correlation P-value. The first component of each module was obtained by singular value decomposition, and the hub bacterium of each module was that with the highest correlation strength with the first component.

### Bacterial Gene Prediction and Functional Annotation

The genome references of group-specific species and hub species were downloaded from the UHGG database. The non-host sequence of each sample was aligned to the bacterial genome reference to filter the specific bacterial sequence using bowtie2. The genome assembly and functional annotation of each bacterial species in each sample were assessed as described previously, except that the coverage of omixer-RPM was 0.66. The bacterial genes were predicted by salmon (version 0.15.0) (Patro et al., 2017) using *de novo* contigs as the reference, and the gene count matrix of each bacterium in each sample was transformed into transcripts per million (TPM). The differential gene expression (DEG) of each species among the groups was identified by the limma R package with logFC cutoff 1.5 and FDR <0.05.

### Statistical Analysis

All statistical analyses were performed using R software, version 3.6.3 (The R Foundation for Statistical Computing, http://www.rproject.org/). Continuous variables between groups were compared by Student’s t-test, one-way analysis of variance (ANOVA) with *post hoc* pairwise Bonferroni tests, or the Wilcoxon rank-sum test. The gender between patients and controls was compared by the chi-square test, and the age was compared using the student’s t-test. Normality and homogeneity of variance were assessed by the Shapiro test and Bartlett test *via* R function, respectively. The correlations between continuous variables were evaluated by Pearson’s correlation analysis. Volcano plots, box plots, dot plots, and bar plots were drawn using ggplot2 or corrplot R packages.

## Results

### Gut Microbiota Diversity and Composition Alteration in Patients and Healthy Controls

**Figure 1:**
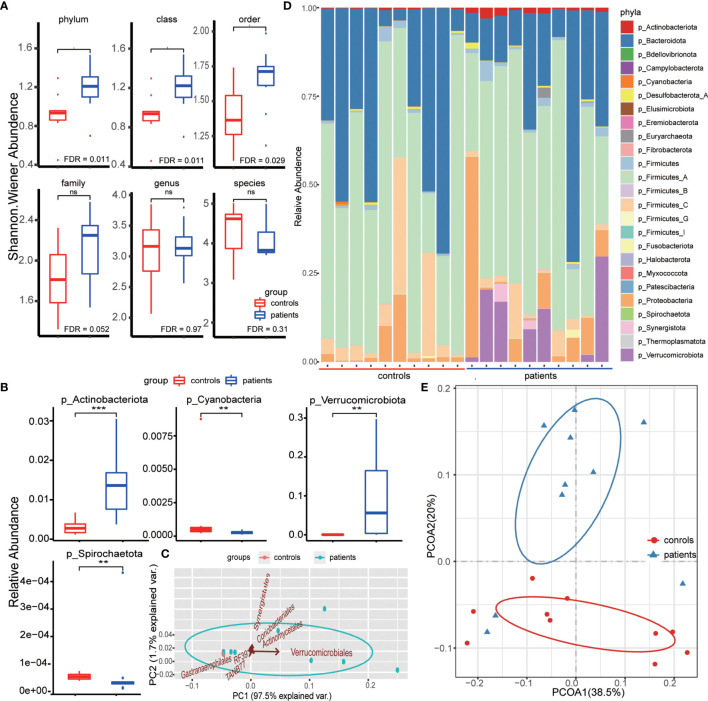
**(A)** Comparison of diversity of each taxonomic level between patient and control groups. The taxonomic indexes and the Shannon Wiener indexes were compared by the Wilcoxon rank-sum test. **(B)** Differential phyla between groups identified by the Wilcoxon rank-sum test based on the relative abundance of bacterial phyla. ** means FDR < 0.01, *** means FDR < 0.001. **(C)** Principal component analysis (PCA) based on the relative abundance of differential orders between groups. The arrow direction represents the correlation between the phyla relative abundance and the principal component, and the arrow length indicates the contribution of corresponding taxonomic order in discriminating patients and controls. **(D)** Phyla composition of each sample. The bar length indicates the relative abundance of each phyla composition, and the total bacterial composition is 1. **(E)** Principal coordinate analysis between two groups based on the weighted unifrac distance. The ellipse represents the core area added by the group according to the default confidence interval. ns means no statistical significance.

No significant difference was detected in consciousness at admission (Glasgow coma scale (GCS), P=0.210), gender (P=0.648), or age (P=0.224) between patients and controls (**Table S1**). After annotating the metagenomic sequence data and removing non-bacterial species, we identified 25 phyla, 35 classes, 86 orders, 272 families, 1386 genera, and 4539 species (**Table S2**). The richness of each sample reached the horizontal asymptote that the data comprised almost all bacteria of each sample, while few remained undetected (**Figure S1A**). The comparison of the richness at each level did not reveal any significant difference between patients and controls (**Figure S1B**). However, when introducing the Shannon index that considers both the number and abundance of species simultaneously to estimate the intragroup alpha diversity, we found additional phyla, classes, and orders in the patient group (**Figure 1A**). This indicated that the gut bacterial transformation in IPH patients could be attributed to the altered gut bacteria at high taxonomic levels, and newly dominant bacterial phyla could be propagated to complement the gut bacteria diversity such that no significant difference was detected between the groups at low taxonomic levels, including family, genus, and species. Although the composition percentage of bacterial phyla varied among samples, we found that the relative abundance of four bacterial phyla, *Actinobacteriota*, *Cyanobacteria*, *Spirochaetota*, and *Verrucomicrobiota*, differed between the groups obviously, among which the difference in *Actinobacteriota* and *Verrucomicrobiota* could be directly identified based on the abundance distribution histogram (**Figures 1B, C**).

To evaluate the contribution of bacteria in these four bacterial phyla to discriminate patients from healthy individuals, we first calculated the LDA scores using LEfSe analysis. This score assessed the impact of significantly different species at each taxonomic level and evaluated the discrimination power at the order level (**Table S3**). Except for no difference in the bacteria of *Spirochaetota* phylum, as assessed by LEfSe analysis, the other three phyla, formerly defined as differential bacterial phyla, had group-specific bacteria with statistical significance at each taxonomic level (**Figure S1C**). Herein, we obtained six bacterial orders with statistical significance (FDR <0.05), consisting of *Actinomycetales* (phylum *Actinobacteriota*), *Coriobacteriales* (phylum *Actinobacteriota*), *Gastranaerophilales* (phylum *Cyanobacteria*), *RF39* (phylum *Firmicutes*), *TANB77* (phylum *Firmicutes*), and *Verrucomivrobiales* (phylum *Verrucomicrobiota*). Among these, *Verrucomivrobiales* (phylum *Verrucomicrobiota*) played a key role in IPH patient discrimination (**Figure 1C**), which was consistent with the bacterial phyla composition of the patient group (**Figure 1D**). To further evaluate the discrimination of the bacterial species in IPH patients, we calculated the weighted unifrac distance that considered the evolutionary correlation of bacteria between every two samples, and 20 samples were clustered into two groups by PCoA. The groups clustered through the weighted unifrac distance were similar to those distinguished by disease status (**Figure 1E**).

### Functional Alteration in the Gut Microbiota of IPH Patients

**Figure 2:**
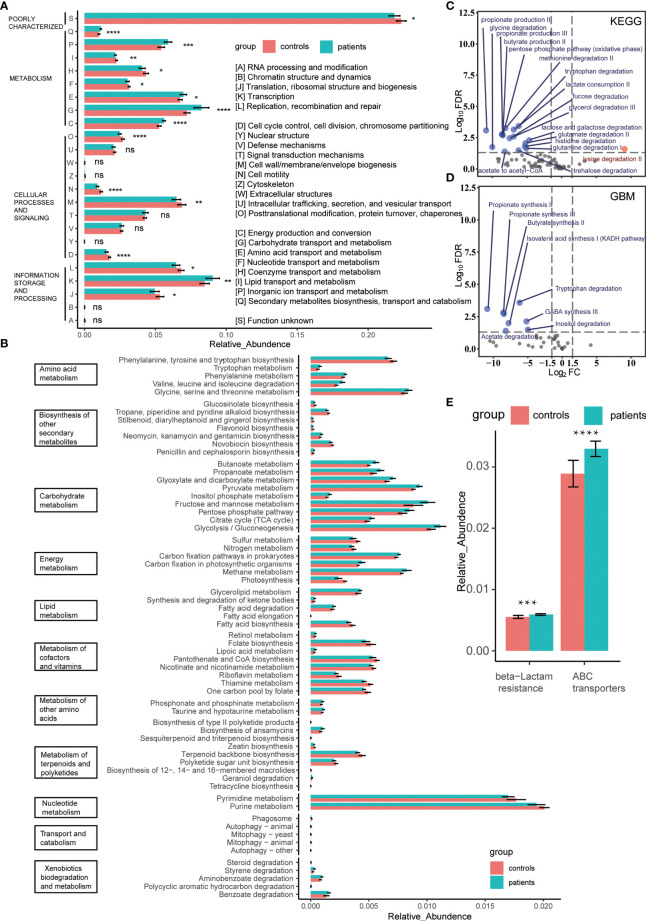
**(A)** Comparison of proteins annotated by the COG database between groups. The relative abundance of protein orthologs in each catalog was compared by the Wilcoxon rank-sum test. * means FDR < 0.05, ** means FDR < 0.01, *** means FDR < 0.001, **** means FDR < 0.0001. **(B)** Bacterial metabolic activity comparison between groups based on the relative abundance of protein orthologs annotated by the KEGG database. All FDRs of metabolic pathways were < 0.05. **(C, D)**. Volcano plot of the KEGG modules or the GBM modules enriched by KEGG protein orthologs, modules with FDR < 0.05 and |log2FC| > 1.5 were identified as the differential modules. The blue and red represent the patient group and controls, respectively. **(E)** Comparison of antibiotic resistance-related pathways between patients and controls. The relative abundance of protein orthologs that participated in the pathways was compared using Wilcoxon rank-sum test. *** means FDR < 0.001, **** means FDR < 0.0001. ns means no statistical significance.

The functional annotation results could be divided into 24 categories based on sequence similarity by aligning the data to the COG database designed for orthologous groups of proteins (**Figure 2A**). According to the biological process, these 24 categories were summarized into four modules: metabolism, cellular processes and signaling, information storage and processing, and poor characterization. Among these, metabolism was a diverse module since the comprising categories varied significantly between the groups. The comparison of the metabolic processes between groups revealed that the capacity of energy production and conversion is increased in the patients’ gut bacteria, consuming excess carbohydrates and amino acids, while the lipid metabolism capacity was decreased slightly. Simultaneously, due to the increased taxonomic diversity and transcription activity of the patients’ gut bacteria, the biosynthesis of the second metabolite was active (**Figures 1A**, **2A** and **Table S4**). In order to further clarify the difference in the metabolic pathway between groups, we introduced the KEGG database and subdivided the metabolic module into 11 specific clusters (**Figure 2B**). We observed that the two groups had significant differences in the overall metabolic pathways (**Figure S2A**). The advantages of energy metabolism of patients’ gut bacteria were manifested as carbon fixation and methane metabolism. In addition to the biosynthesis of aromatic amino acids, the metabolism of amino acids was active in the patient group. Regarding lipid metabolism cluster, the gut bacteria of patients degraded lipids, such as fatty acids, ketone bodies, and glycerolipids, while the healthy people’s gut bacteria were more active in biosynthesis. Moreover, all the identified differential carbohydrate metabolic pathways were active in patients, which explains the capability of increased short-chain fatty acid production, including acetate, propionate, and butyrate, in patients’ gut bacteria (**Figures 2C** **, D**) (Sonnenburg and Bäckhed, 2016). Conversely, the metabolism of cofactors, vitamins, and nucleotides, including pyrimidines and purines, was more active in the controls. Next, we used the CAZy database to identify the bacterial carbohydrate metabolism-related enzymes that contributed to the carbohydrate metabolic difference between groups and found that the carbohydrate biosynthesis-related enzymes were enriched at healthy controls’ gut bacteria (**Figure S2B**). On the other hand, after KEGG annotation, we found that the antibiotic resistance-related pathways, especially the resistance to β-lactam, were enriched in the patients’ gut bacteria despite no previous antibiotic exposure for at least 3 months, which was consistent with the antibiotic resistance gene upregulation annotated by the CARD database (**Figure 2E** and **Figure S2C**). To evaluate the adverse impact of gut bacteria, we aligned two groups’ non-host data to the VFDB. The virulence factors secreted by the dominant genus, such as *Klebsiella* (expansion induces colitis in mice) (Garrett et al., 2007), *Escherichia*, and *Clostridium*, were elevated in the patients’ gut bacteria, indicating a high taxonomic diversity of the corresponding dominant genus in the patients’ gut (**Figure S2D**). The virulence factors were enriched explicitly in the type VI secretion system (**Figure S2E**). Due to the function of the microbial complex of mediating interstrain killing (Chatzidaki-Livanis et al., 2016), the bacteria transformation from the non-dominant genus to the dominant genus occurred in patients’ gut as indicated by the altered bacterial secretion diversity in virulence factors between the two groups (**Figure S2D**).

### Functional Alteration of Group-specific Species Contributes to the Functional Difference Between Groups

**Figure 3:**
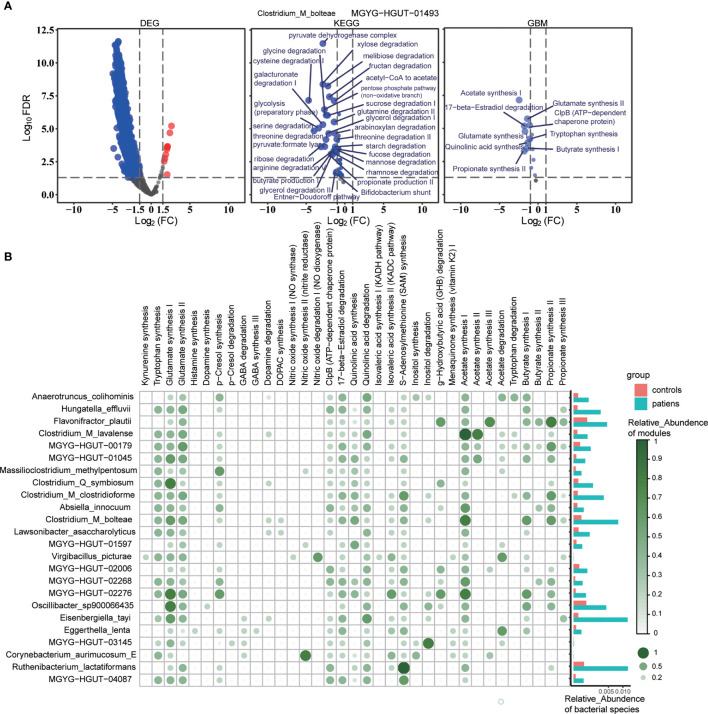
**(A)** Volcano plots of DEGs, the KEGG modules, and the GBM modules of one specific species of patient group, *Clostridium bolteae*. DEGs with FDR < 0.05 and |log2FC| > 1.5 and modules with FDR < 0.05 were identified as differential genes or modules. The modules with |log2FC| > 1 are marked in the figure. The blue and red represent the patient group and the control group, respectively. MGYG-HGUT-01493 is the ID of this species in the UHGG database. **(B)** Dot plot of neuroactive compound metabolism (GBM) of patient group-specific species. The dots mean that species have the metabolic pathway and the bars next to the dot plot mean the relative abundance of this species in each group. The color and size of the dots mean the relative abundance of the metabolic pathway. Each row represents the species, and the column represents the GBM pathways.

A significant positive correlation was established with the environment, which indicates a solid environmental selection (Lozupone et al., 2012). To identify the bacterial species selected by disease state, we associated 4539 bacterial species with the groups and defined the species with positive coefficients and FDR <0.05 as the group-associated species (**Table S5**). The intersection of the differential species obtained from the LEfSe analysis on the group-associated species retrieved the group-specific species (26 for controls and 24 for patients) (**Table S6**). The metabolic activity of group-specific species was much higher in the related group, especially the patient group, and the differential expressed gene (DEG) characteristics of these species were consistent with the metabolic alteration trends (**Figure 3A** and **Figures S3**, **S4**). GBMs were designed to characterize the neuroactive potential of gut microbiota corresponding to a single neuroactive compound production or degradation process. Then, we determined whether the GBMs, present in each group-specific species, varied significantly between patients and controls. The patients’ group-specific species produced neuroactive compounds and biosynthesized short-chain fatty acids, especially propionate and butyrate, in the patients’ group-specific species (**Figure 3B**, **Figure S5A**). The comparison among the GBMs’ metabolic activity of all the 50 group-specific species between groups revealed a high neuroactive compounds’ metabolic capability of each group-specific species in the associated group, consistent with the KEGG metabolic pathways and DEG characteristics of each species (**Figure 3A** and **Figures S6**, **S7**). The phylum *Verrucomicrobiota* was identified specific bacterial phylum of the patient group, and we selected all species in this phylum and estimated their metabolic feature. The species of phylum *Verrucomicrobiota* were specialized in acetate synthesis and the degradation of carbohydrates and mucin, which might elevate higher carbohydrate metabolism in the patient group (**Figure S8**).

**Figure 4:**
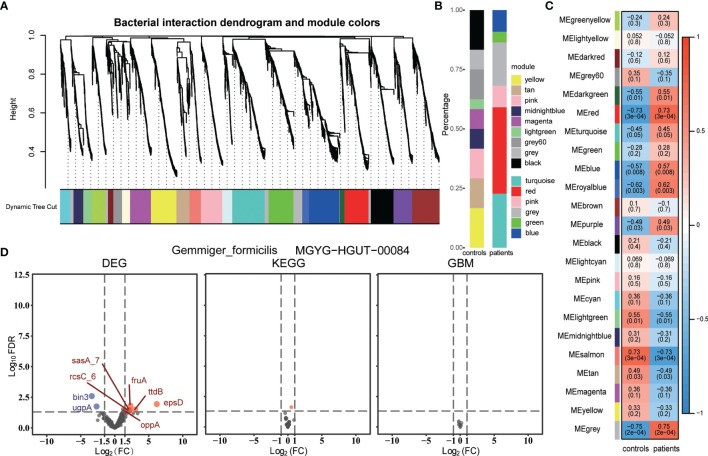
**(A)** Bacteria interaction modules identified by WGCNA. **(B)** Percentage of each WGCNA module bacteria in the group-specific species. **(C)** Pearson’s correlation between bacterial WGCNA modules and groups. Each cell contains the coefficient, from -1 to 1 and the P-value. **(D)** Volcano plots of DEGs, the KEGG modules, and the GBM modules of one WGCNA module hub bacterium, *Gemmiger formicilis*. DEGs with FDR < 0.05 and |log2FC| > 1.5 and modules with FDR < 0.05 were identified as differential genes or modules. The blue and red represent the patient group and the control group, respectively. MGYG-HGUT-00084 is the ID of this species in the UHGG database.

Next, we assessed whether the non-differential bacterial species had the same metabolism and transcription features as the group-specific species. Firstly, we constructed a bacterial weighted correlation network and obtained 22 bacterial interaction modules (**Figure 4A**). Then, we correlated these bacterial modules with groups and obtained module significance and hub bacteria of each module (**Figure 4C**, **Table S7**). Former identified group-specific bacteria gathered in the modules were significantly associated with the corresponding group (**Figure 4B**). The hub bacteria of the light-yellow module, *Gemmiger formicilis*, was selected for the least correlation coefficient of either group. However, no significant difference was observed in the KEGG and GBM metabolic modules between the two groups, while only some differentially expressed genes were detected (**Figure 4D**). Simultaneously, we compared the metabolic modules of other hub bacteria but did not find any significant differences between the groups (**Figure S9**). The missing hub bacteria metabolic comparison did not identify any meaningful metabolic pathways.

## Discussion

The gut-brain axis enables the communication between the central nervous system (CNS) and enteric nervous system (ENS) sponsored by gut bacteria change (Hanscom et al., 2021). The gastrointestinal dysfunction occurs in patients after cerebrovascular accidents (Iftikhar et al., 2020), which might be partially caused by gut microbiota imbalance. Meanwhile, hypothalamic-pituitary-adrenal (HPA) axis and its associated hormones, like noradrenaline increased after brain trauma, influence the gut bacterial proliferative ability and pathogenicity (Sudo, 2014; Houlden et al., 2016). Gut microbiome composition and diversity could be affected by intestinal motility, transit, barrier integrity, and different factors’ secretion modulating by ENS activity, mediated by CNS input. The neural signal-mediated gut activity, together with the activated immune system and endocrine change after IPH, could be the potential mechanism of IPH patients’ gut bacteria alteration. (Kashyap et al., 2013; Gensollen et al., 2016; Zhu et al., 2018; Hanscom et al., 2021). Previous studies have proved that acute brain injury induces specific changes in the mice gut microbiota that affects the outcome in mice (Benakis et al., 2016; Houlden et al., 2016; Mazarati et al., 2021). However, whether gut microbiota dysbiosis occurs in patients with acute cerebrovascular events remains unknown. Herein, we performed metagenomic shotgun sequencing on fecal samples of 10 IPH patients and 10 healthy controls with corresponding characteristics. The gut bacteria composition of patients with IPH significantly changed within 7 days, among which the phylum *Verrucomicrobiota* accumulated in the patients’ gut. Acute brain injury, such as IPH induces gut microbiota transformation from the up taxonomic levels and forms a new bacterial interaction network to compensate the bacterial alpha diversity at the low taxonomic levels in the gut. Phylum *Verrucomicrobiota* enrichment can specifically distinguish IPH patients from healthy people, similar to the finding that family *Verrucomicrobiaceae* is enriched in the gut of mice suffering traumatic brain injury (Opeyemi et al., 2021). This phylum, *Akkermansia muciniphila*, a next-generation probiotic (O'Toole et al., 2017), is the main differential species (**Table S3**). Previous studies have shown that its high relative abundance is associated with a healthy metabolic status by improving the intestinal barrier and alleviating gut inflammation (Dao et al., 2016; Plovier et al., 2017; Tang et al., 2019; Wang et al., 2020). In the current study, the increased relative abundance of *Akkermansia muciniphila* in the patient group indicated that the gut microbiota could adjust its composition, increasing probiotic abundance and producing beneficial neuroactive compounds, such as short-chain fatty acids (**Figure S8**) to decrease the adverse impact of acute cerebrovascular events. Next, we proposed that bacterial composition transformation is the gut microbiota feedback to the adverse events, which also involves functional alteration of gut microbiota. The high metabolic state is the primary functional feature of the patients after IPH, consuming excessive carbohydrates, lipids, and amino acids for energy production and producing beneficial neuroactive compounds to alleviate the damage, accompanied by low activity in the cellular processes as cellular structure biogenesis. Otherwise, accompanied by high abundance, the dominant pathogenic bacteria, including *Klebsiella*, *Escherichia*, and *Clostridium*, cause disease by secreting virulence factors in patients’ gut, although the total virulence factor diversity was less than that in the controls’ gut bacteria. The increased relative abundance and virulence factor diversity of these three dominant pathogenic bacteria indicated the bacterial selection of the patients’ disease state, suppressing other pathogenic genera and specifically allowing the selected pathogenic bacteria, *Klebsiella*, *Escherichia*, and *Clostridium*, to gain abundance in order to secrete various virulence factors and unselected pathogenic bacteria to lose diversity, succumbing to the dominant bacteria colonization. IPH also increases the antibiotic resistance of the gut bacteria, especially to beta-lactam, which deserves further exploration. Recent studies reported that antibiotic therapy was associated with gut bacterial diversity absence and might be potentially harmful (Ravi et al., 2019; Arulkumaran et al., 2020; Celorrio et al., 2021). Combined with our findings, the alteration of gut bacteria antibiotic resistance after IPH needs to be considered when selecting the drugs for preventive antibiotic therapy (Simon et al., 2020).

The metabolic and transcriptional activity alteration of the gut bacteria might be inconsistent with the composition change (McNulty et al., 2011). To decipher whether the metabolism difference in IPH patients is associated with the modification in the bacterial composition, we recruited representative bacteria for further analysis from enterotype, a stable bacterial interaction network identified in the human gut whose alteration is associated with a long-term diet intervention (Arumugam et al., 2011; Wu et al., 2011). Next, we identified the group-specific species of each group and the non-differential species. Owing to the bacteria influencing the human body by the network (Mac Aogáin et al., 2021), we introduced WGCNA to construct the bacterial interaction network during non-differential species selection that has an indirect influence while measuring the inter-species interactions. Then, we chose the hub bacteria as the representative due to their central position in the interaction network. In our cohort, not only the gut bacteria composition ratio changed significantly, the metabolic activity of the corresponding group-specific species varied between groups contrary to the WGCNA module hub bacteria even when the module significantly correlated with one of the groups. Additionally, the transcription activity of the group-specific species boosted in the corresponding group, suggesting that metabolism alteration was due to active gene transcription. This difference between patients and healthy people is attributed to group-specific species activity change, including transcription, rather than that of the non-differential species in IPH patients. Furthermore, the neuroactive compounds were diversified in the patients’ specific-group species. In addition to the common compounds synthesized by each species, such as glutamate and quinolinic acid, the patient’s group-specific species produce additional beneficial molecules, such as butyrate and propionate, which alleviate deteriorate factors, like inflammation state and metabolic disorders, thereby improving the functional outcomes (Vipperla and O'Keefe, 2012; Opeyemi et al., 2021). Therefore, we hypothesized that the altered gut bacterial composition and function is another mechanism after suffering IPH through which the gut microbiota transformation might alleviate the adverse effects and promote neural recovery, thereby improving the outcomes in patients. However, the prognostic value of gut bacteria is yet to be explored.

Nevertheless, one of the limitations that affect the results of metagenomics is the resolution. Previous studies on the gut microbiota selected 16S amplicon sequencing. This sequencing strategy that analyzed the bacterial V3-V4 region of the 16S RNA gene has a limited resolution in bacterial species identification, capturing reliable taxonomic classification only at the genus level (Matias Rodrigues et al., 2017). However, several studies suggested that many taxonomic and functional associations are present only at the species level (Costea et al., 2017; Lloyd-Price et al., 2017). Thus, we conducted the metagenomics shotgun sequencing of the whole genome of the fecal microbiota, even if the species were inaccessible by cultivation, to improve taxonomic resolution and annotate the gut bacterial function in each group. We also introduced the UHGG database, the most comprehensive microbial public collection comprising 204,938 non-redundant genomes from 4,644 gut prokaryotes (Almeida et al., 2021), to conduct the taxonomic annotation. By matching with this database, we obtained an accurate characterization of the taxonomic and functional repertoire of the gut microbial ecosystem. The gut microbiota mainly was composed of two dominant bacterial phyla, *Firmicutes* and *Bacteroidota*, with other subdominant phyla including Proteobacteria, *Actinobacteriota*, and *Verrucomicrobiota*, which was similar to previous results (Qin et al., 2010). Nonetheless, the present study still has some limitations. Since it is difficult to predict the occurrence of IPH, we collected fecal samples from the corresponding healthy controls instead of the patients’ sample before the disease occurs. Inevitably, we missed the potential species that altered before the hemorrhage and some confounding factors, such as the genetic background, diet, and living habits, between groups. In clinical practice, patients suffering from IPH were recommended a fast or liquid diet, which differs from healthy control. The diet difference between groups is a potential confounder to bacterial composition. However, some studies pointed out that short-term diet intervention is less likely to affect bacterial composition (Wu et al., 2011; O'Keefe et al., 2015). The participants selected in the study were native residents from the same region, and the samples collected were at the same season, thereby reducing the impact of time and space. To obtain a valid group-specific species list and reduce the impact caused by sample size limitation, we conducted a parallel maaslin correlation analysis apart from the LEfSe analysis. The species selected by both analyses were identified as group-specific species. Due to the extremely low relative abundance of the phylum *Verrucomicrobiota* in controls and the limited sample number, which led to an insignificant FDR of corresponding species, we did not obtain any species in this phylum identified as group-specific species of patients even though the relative abundance of phylum *Verrucomicrobiota* altered between groups. Similarly, we filtered out some of the differential species between groups by this correlation analysis, which may be the potential group-specific species, such as that in phylum *Verrucomicrobiota*, or the false positive specific species caused by the random extreme abundance disparity due to the sample size limitation, thereby necessitating a valid group-specific species list for further analysis. Subsequently, we found an obvious alteration in the group-specific species than the non-differential species, indicating the pivotal function of these group-specific species in the patients’ gut.

## Conclusion

In conclusion, gut bacteria transformation is a potential mechanism influencing the outcome of patients. The current findings showed taxonomic composition and functional changes in gut bacteria in patients after IPH. The taxonomic composition of patients’ gut bacteria (phylum *Verrucomicrobiota*) altered significantly after suffering from IPH. Moreover, the bacterial functional alterations, including metabolism, antibiotic resistance, and virulence, are attributed to the group-specific species and need to be considered when targeting the gut bacteria during IPH treatment.

## Data Availability Statement 

The datasets presented in this study can be found in online repositories. The names of the repository/repositories and accession number(s) can be found below: National Center for Biotechnology Information (NCBI) BioProject database under accession number PRJNA806955.

## Ethics Statement

The studies involving human participants were reviewed and approved by the Institutional Review Board of Jishou University. The patients/participants provided their written informed consent to participate in this study.

## Author Contributions

ZX and KP designed the study while SS collected samples. The bioinformatics workflow was designed by ZX, assisted by KP, YZ and JG. The manuscript was drafted by ZX. The revision was checked by ZX and JG. CH and XL came up with the study idea and supported the study. All authors contributed to the article and approved the submitted version.

## Funding

This study was supported by The National Key Research and Development Program of China (Grant No. 2016YFC1100605), the National Natural Science Foundation of China (for XL, Grant No. 81770781 and No. 81472594), Special funds for the construction of innovation in Hunan Province (2020SK2062), and the Hunan Science and Technology Innovation Platform and Talent Plan Project (2016SK4007).

## Conflict of Interest

The authors declare that this study was conducted in the absence of any commercial or financial correlations that could be construed as a potential conflict of interest.

## Publisher’s Note

All claims expressed in this article are solely those of the authors and do not necessarily represent those of their affiliated organizations, or those of the publisher, the editors and the reviewers. Any product that may be evaluated in this article, or claim that may be made by its manufacturer, is not guaranteed or endorsed by the publisher.
