# Supplementary material for: Cerebral Intraparenchymal Hemorrhage Changes Patients’ Gut Bacteria Composition and Function
Source: Front Cell Infect Microbiol. 2022 Mar 16;12:829491. doi: 10.3389/fcimb.2022.829491 (PMC8966894; doi:10.3389/fcimb.2022.829491)
Supplement: Supplementary file 1 [file DataSheet_1.pdf]

SUPPLEMENTAL MATERIAL

Cerebral intraparenchymal hemorrhage induces composition and function changes of the patient-specific human gut bacteria species

Zujian Xiong[1,2,3], Kang Peng[1,2], Shaoyu Song[4,5], Yongwei Zhu[1,2], Chunhai Huang[4,5,*], Xuejun Li[1,2,*]

[1]Department of Neurosurgery, Xiangya Hospital, Central South University, Changsha, Hunan 410008, P. R. China
[2]Hunan International Scientific and Technological Cooperation Base of Brain Tumor Research, Xiangya Hospital, Central South University, Changsha, Hunan, 410008, P. R. China
[3]Xiangya School of Medicine, Central South University, Changsha, Hunan, 410008, P. R. China
[4]Department of Neurosurgery, First Affiliated Hospital of Jishou University, Jishou, Hunan, 416000, P. R. China
[5]Centre for Clinical and Translational Medicine Research, Jishou University, Jishou, Hunan, 416000, P. R. China

*** Correspondence:**
Corresponding Author: Chunhai Huang
E-mail: huangchunhai2001@126.com

Corresponding Author: Xuejun Li
E-mail: lxjneuro@csu.edu.cn

Supplemental Figure S1

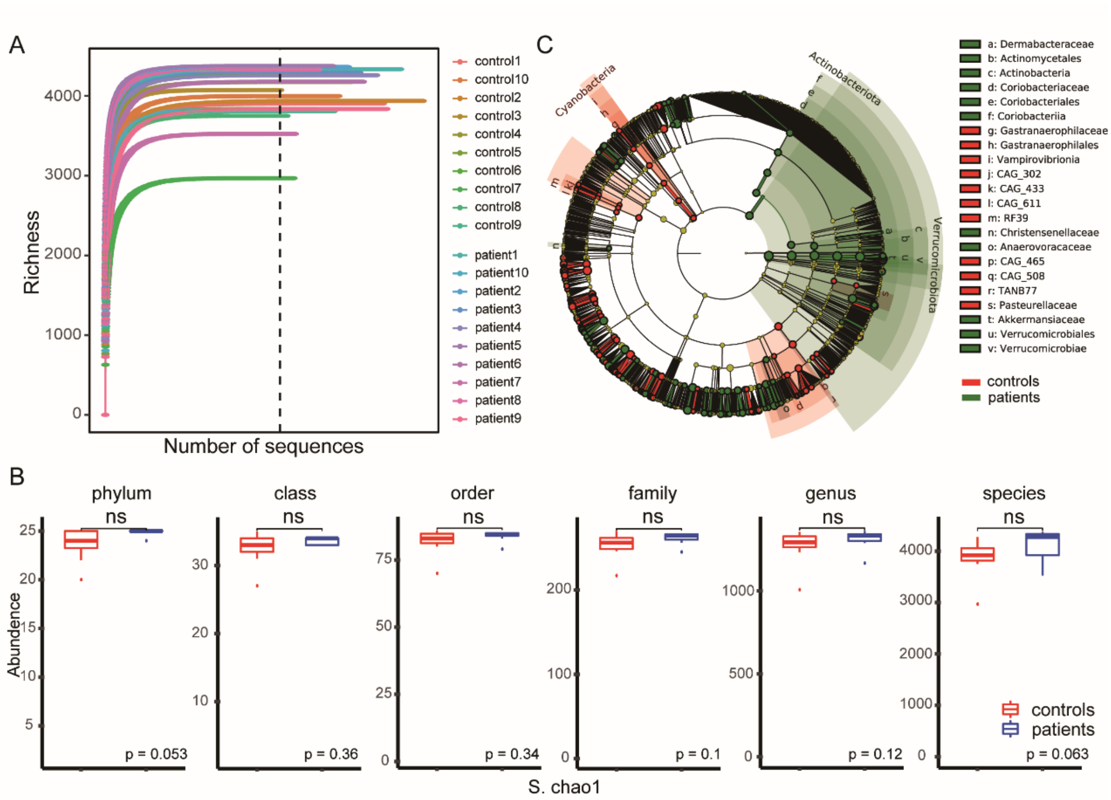

Fig. S1 A, the rarefaction curve of each sample metagenomic data. All curves reach the horizontal asymptote representing fewer species are undetected. B, comparison of taxonomic richness between groups. The taxonomic indexes, the Chao1 indexes, were compared by the Wilcoxon rank-sum test. C, the differential bacteria at each taxonomic level identified by LEfSe between groups. The circles from inside to outside represent the taxonomic level from phylum to species. The red or green dots mean the differential bacteria in controls or patients and the yellow dots mean the non-differential bacteria.

Supplemental Figure S2

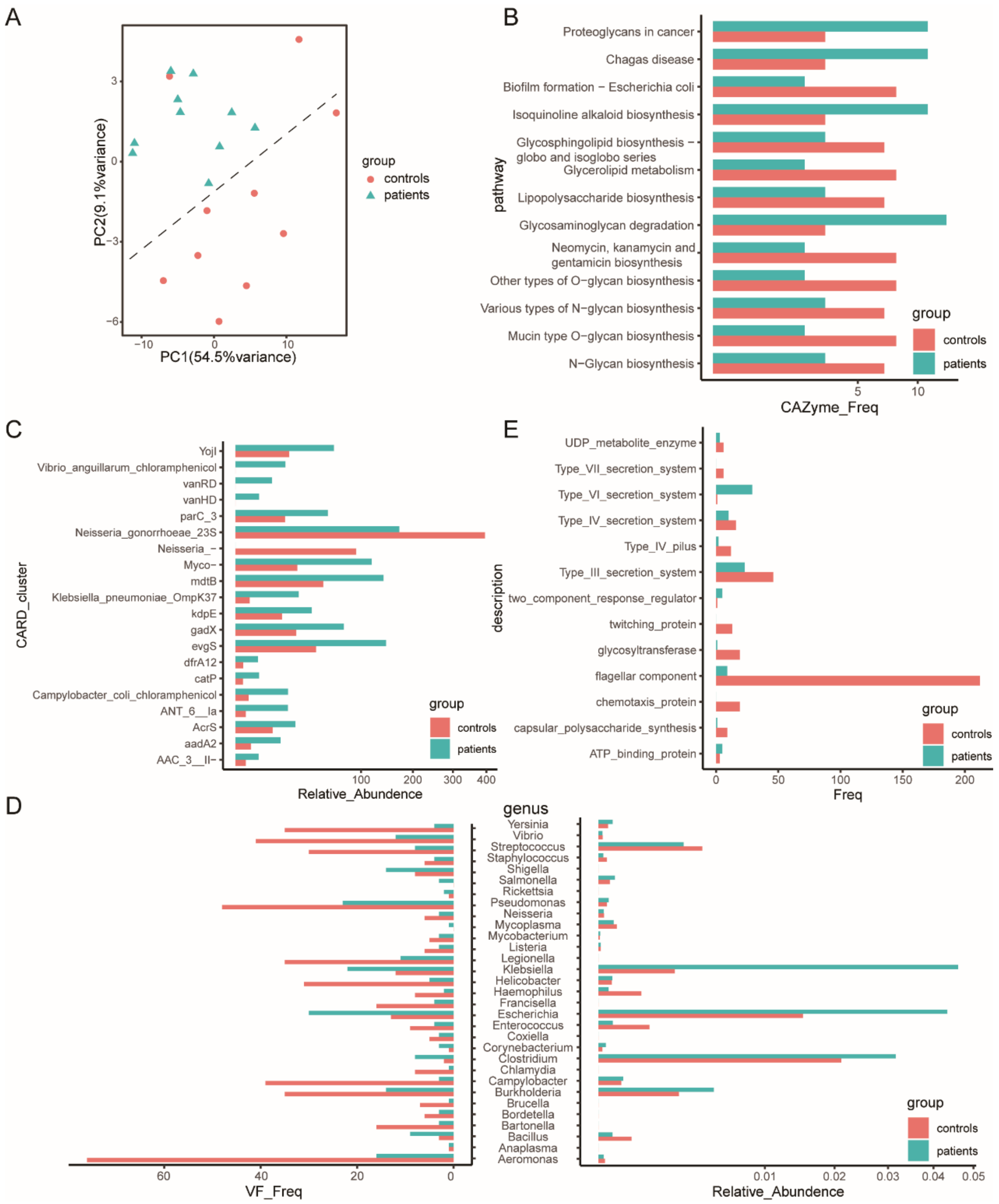

Fig. S2 A, PCA based on the KEGG metabolic pathway abundance. B, the count of differential carbohydrate metabolism-related enzymes with FDR < 0.05 in each carbohydrate metabolic pathway. C, comparison of antibiotic resistance-related genes' relative abundance between group. All FDRs of genes shown in the figure were less than 0.05. D, left bar plot: the count of differential virulence factors with FDR < 0.05 secreted by each genus. Right bar plot: the relative abundance of the corresponding bacterial genus. E, the enrichment of differential virulence factors with FDR < 0.05 between groups. bar length means the count of virulence factor that belongs to the description item.

Supplemental Figure S3

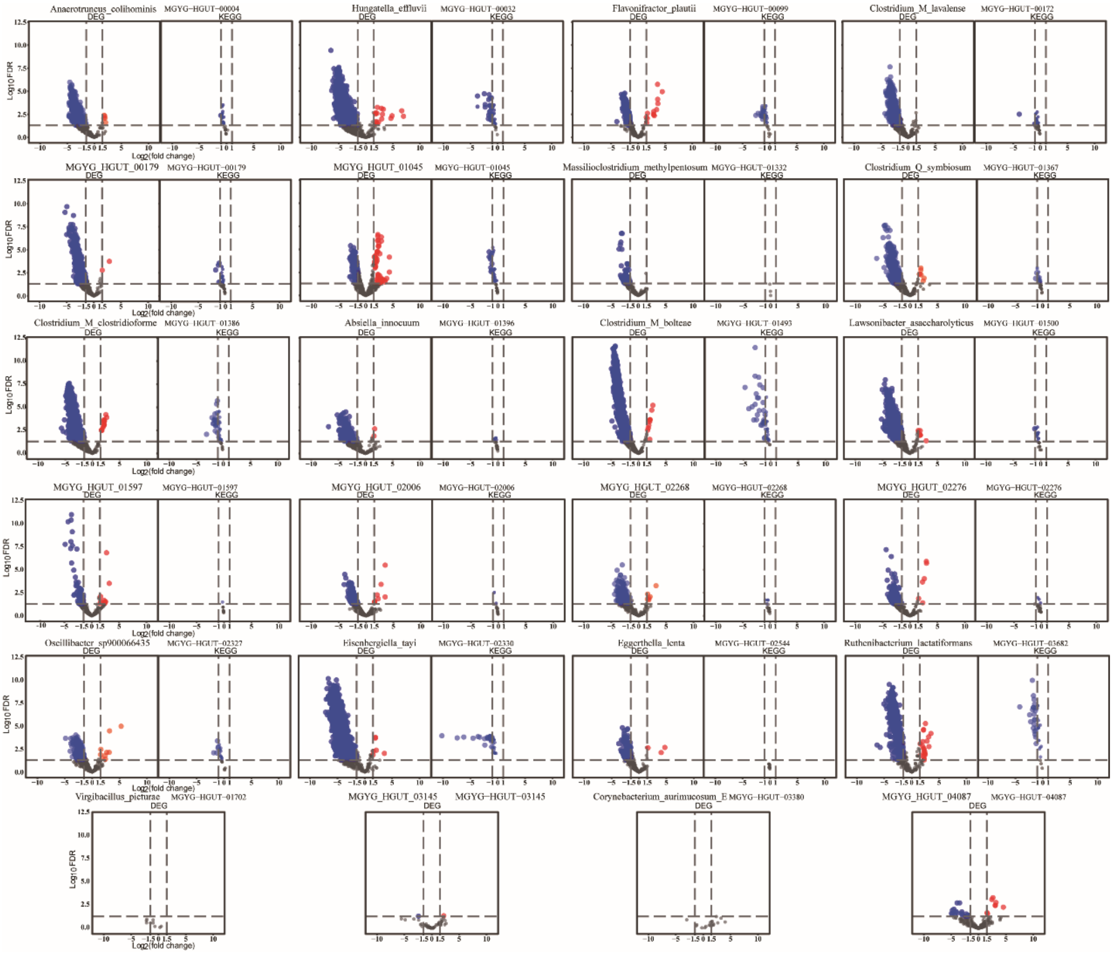

Fig. S3 the volcano plots of differential expressed genes (DEGs) and differential KEGG modules of patient group-specific species. DEGs with FDR <0.05 and |log2FC| >1.5 (x axis) modules with FDR < 0.05 were identified as differential genes or modules. The modules with |log2FC| >1 (x axis) were marked by increasing the dot size in the figure. The blue and red represent the patient group and the control group. MGYG-HGUT is the ID of species in the UHGG database.

Supplemental Figure S4

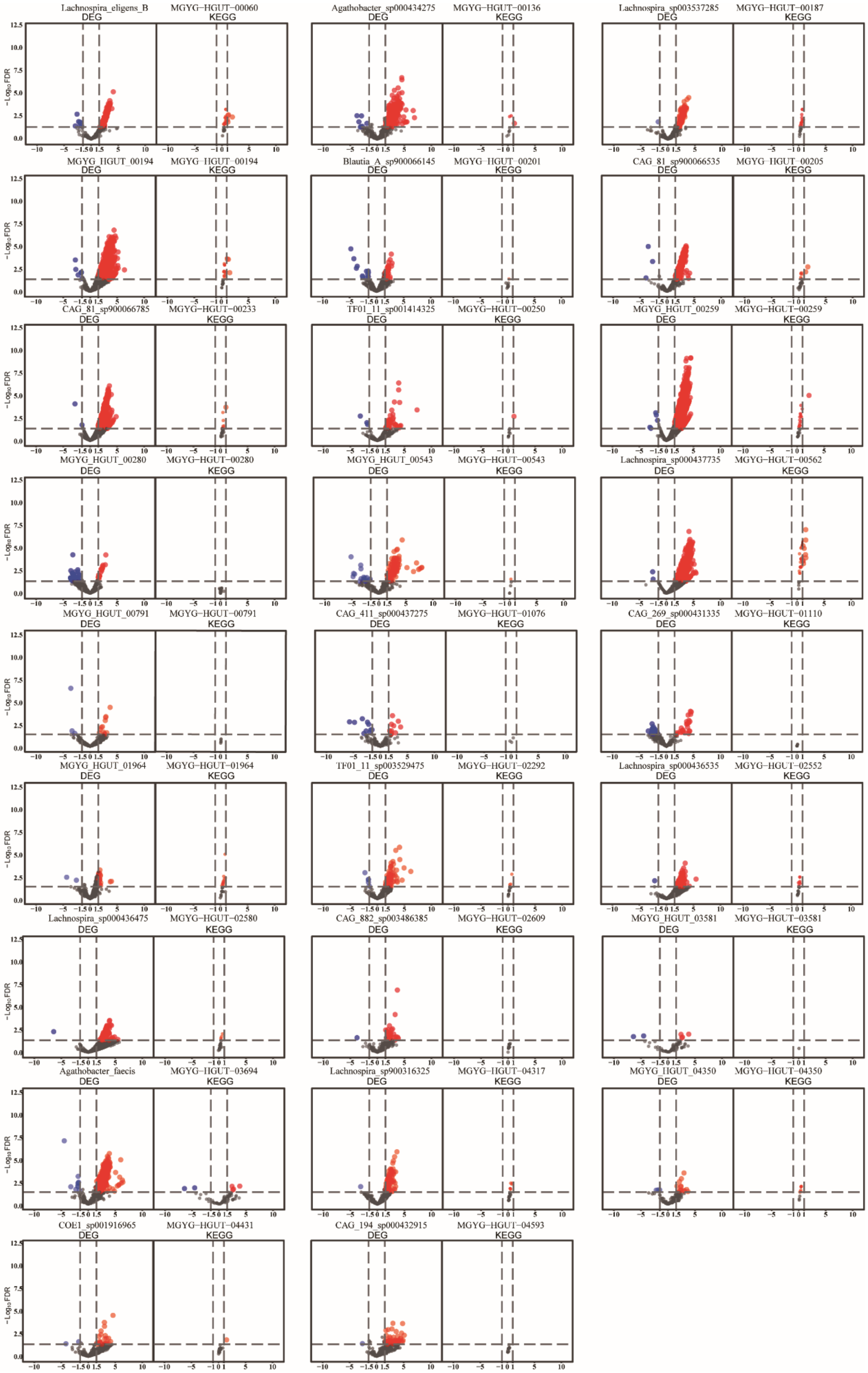

Fig. S4 the volcano plots of differential expressed genes (DEGs) and differential KEGG

modules of control group-specific species. DEGs with FDR <0.05 and |log2FC| >1.5 (x axis) and modules with FDR < 0.05 were identified as differential genes or modules. The modules with |log2FC| >1 (x axis) were marked by increasing the dot size in the figure. The blue and red represent the patient group and the control group. MGYG-HGUT is the ID of species in the UHGG database.

Supplemental Figure S5

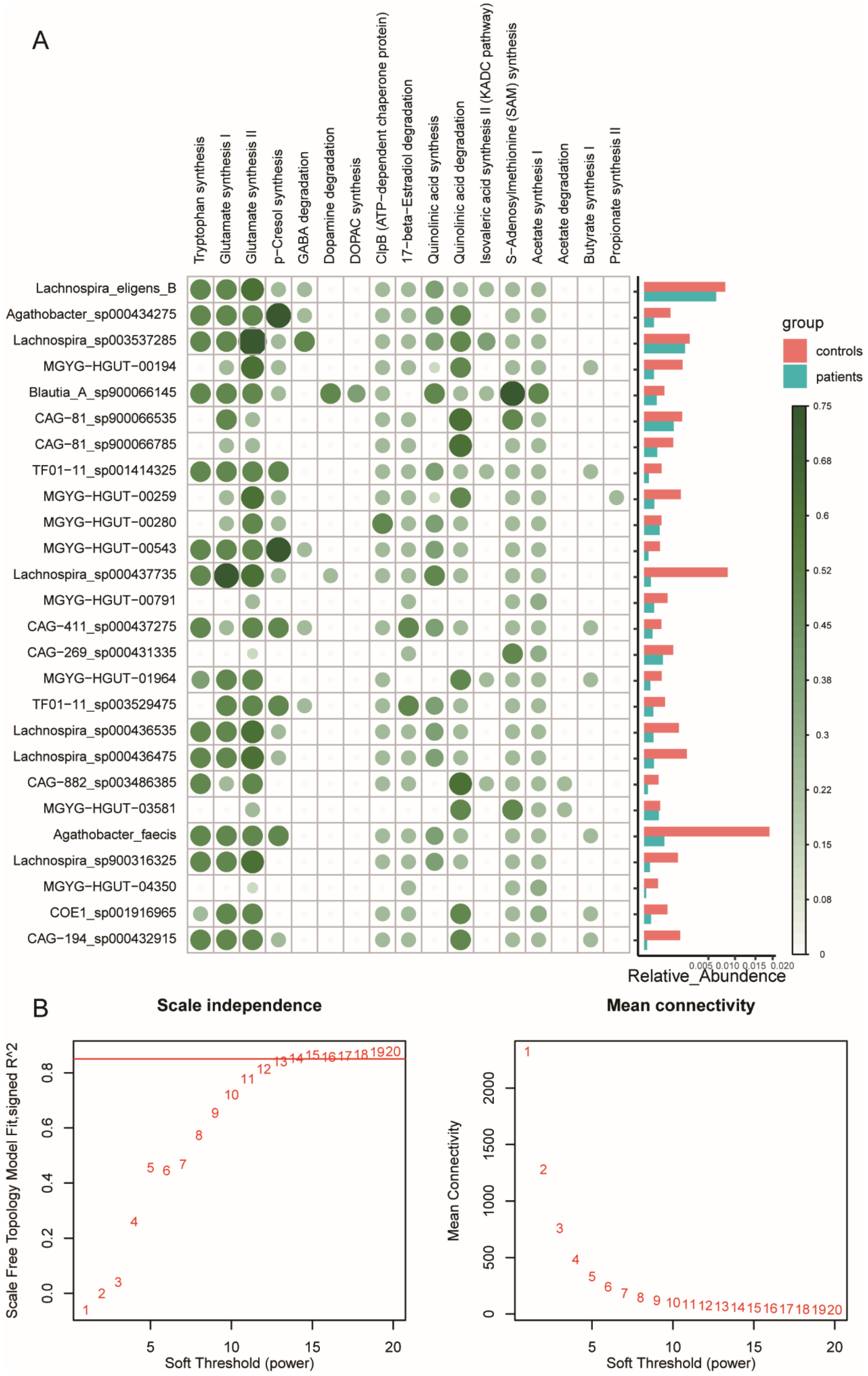

Fig. S5 A, the dot plot of neuroactive compound metabolism (GBM) of control group-specific species. The dots mean that species have the metabolic pathway and the bars next to the dot plot mean the relative abundance of this species in each group. The color and size of the dots mean the relative abundance of the metabolic pathway. B, identification of the soft threshold of network analysis according to the standard of the scale-free network. (power = 14).

Supplemental Figure S6

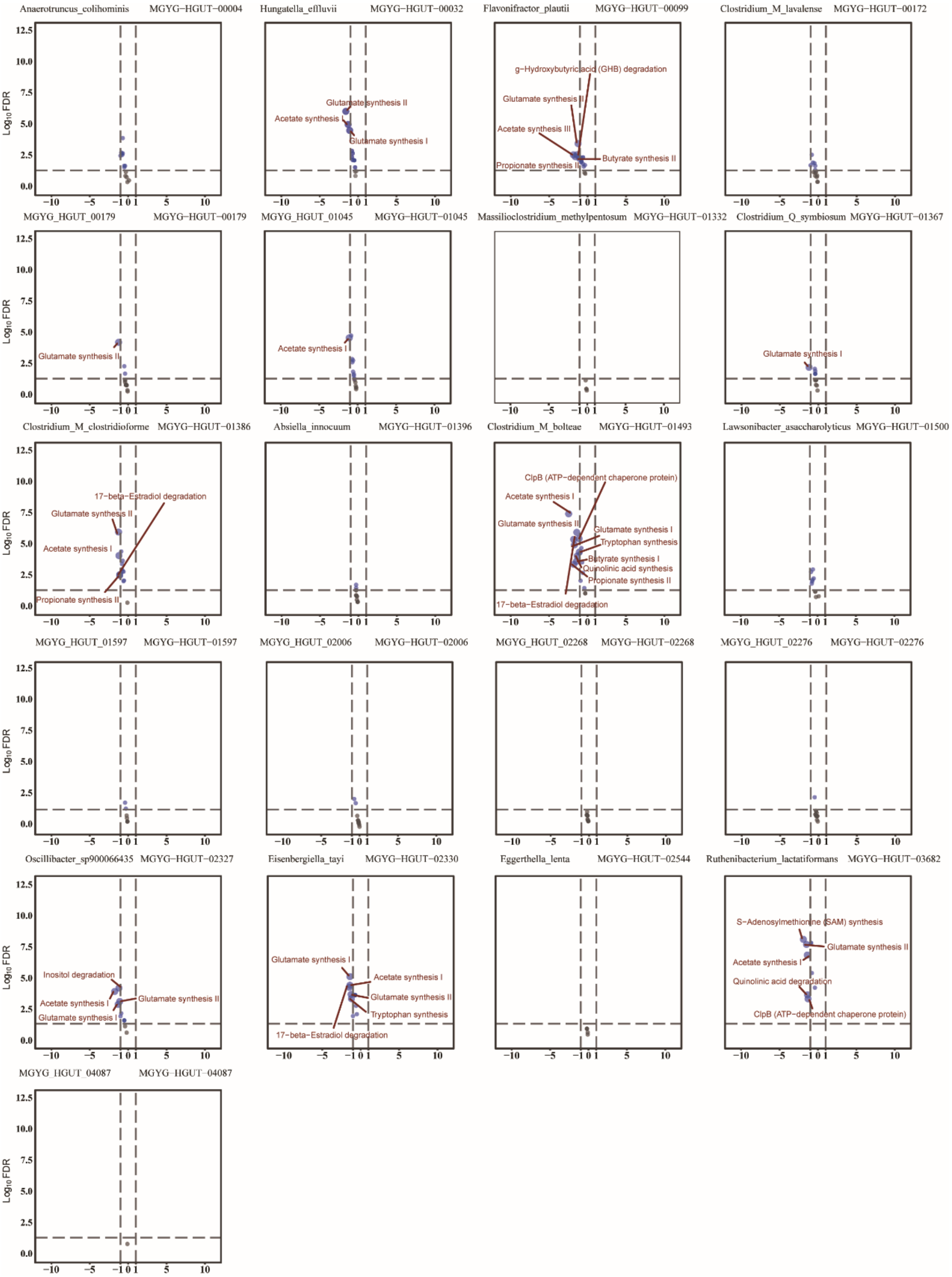

Fig. S6 the volcano plots of differential GBM modules of patient group-specific species. Modules with FDR < 0.05 were identified as differential modules. The modules with |log2FC| >1 (x axis) were marked in the figure. The blue and red represent the patient group and the control group. MGYG-HGUT is the ID of species in the UHGG database.

Supplemental Figure S7

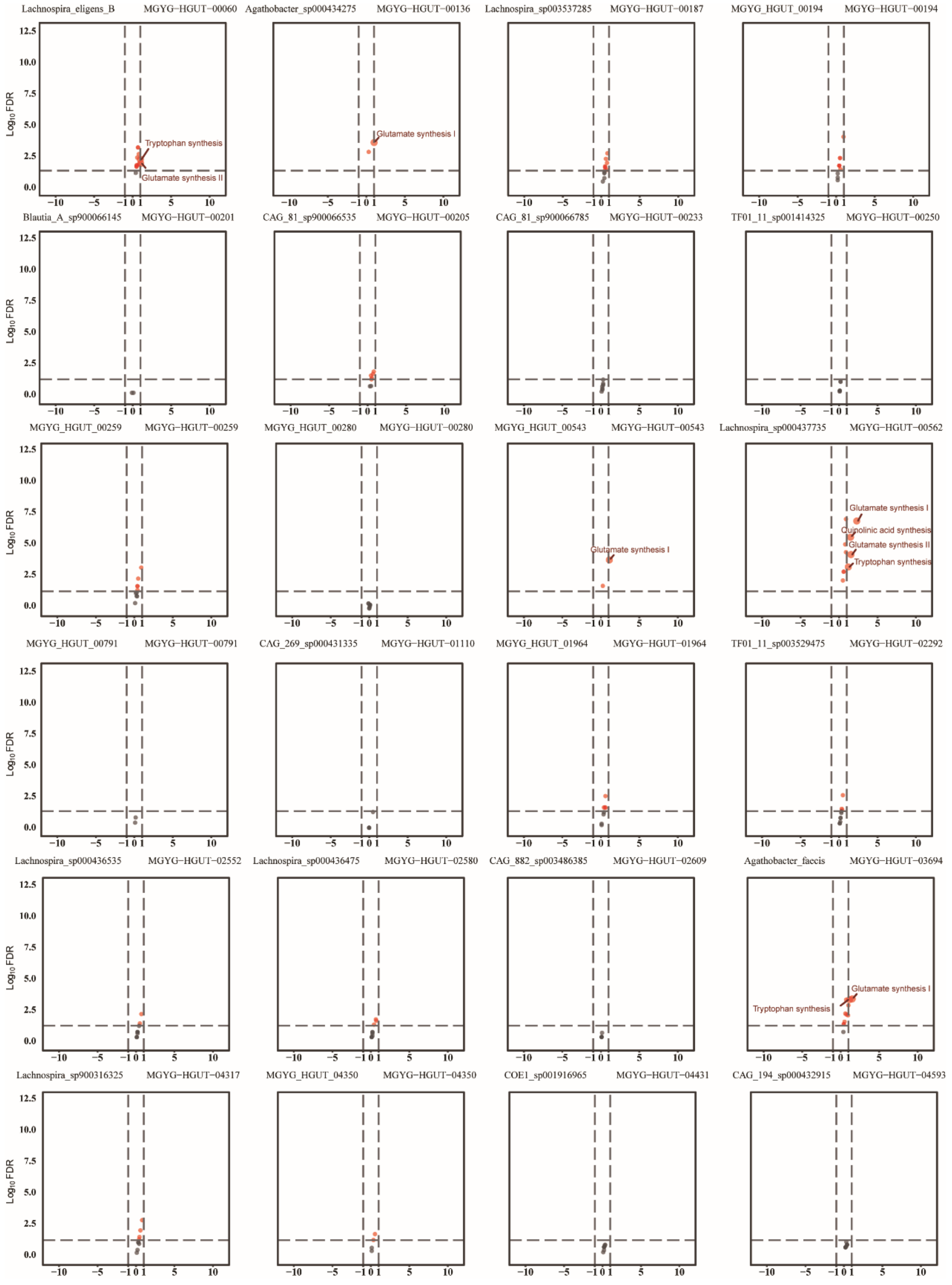

Fig. S7 the volcano plots of differential GBM modules of control group-specific species. Modules with FDR < 0.05 were identified as differential modules. The modules with |log2FC| > 1 (x axis) were marked in the figure. The blue and red represent the patient group and the control group. MGYG-HGUT is the ID of species in the UHGG database.

Supplemental Figure S8

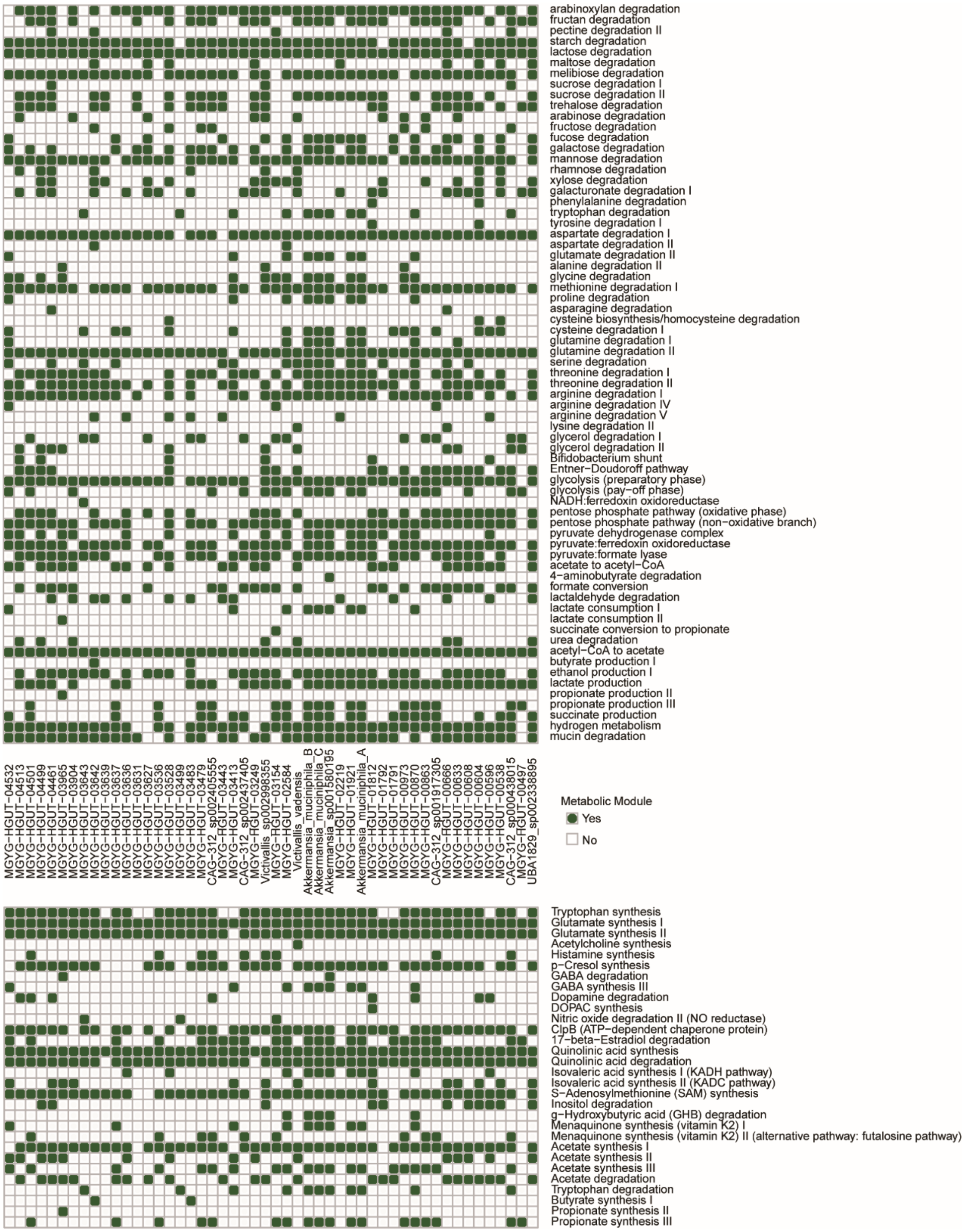

Fig. S8 the dot plot of the KEGG metabolic pathway (upper) and neuroactive compound metabolism (GBM) (lower) of each species in phylum *Verrucomicrobiota*. The dots mean that species have the metabolic pathway.

Supplemental Figure S9

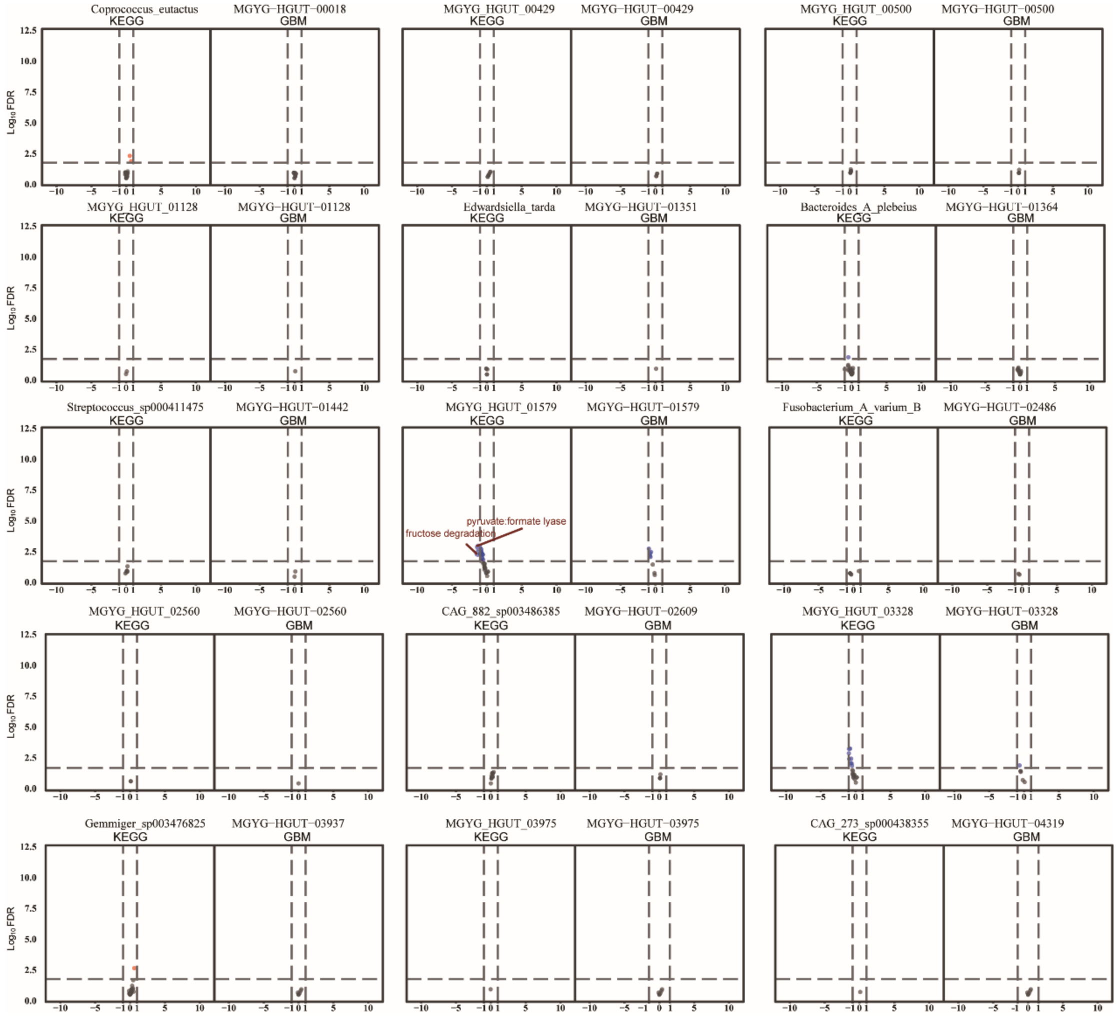

Fig.S9 the volcano plots of differential KEGG modules and GBM modules of WGCNA module hub species. Modules with FDR < 0.05 were identified as differential modules. The modules with |log2FC| >1 (x axis) were marked in the figure. The blue and red represent the patient group and the control group. MGYG-HGUT is the ID of species in the UHGG database.
